# Was Alpha deadlier than wild-type COVID? Analysis in rural England

**DOI:** 10.1007/s15010-022-01787-x

**Published:** 2022-03-05

**Authors:** Julii Brainard, Carlota Maria Grossi Sampedro, Anna Sweeting, Ric Fordham

**Affiliations:** Norwich Medical School, University of East Anglia, Norwich, NR4 7TJ UK

**Keywords:** COVID-19, Risk factors, Epidemiology, Disease outbreak, Deprivation, Mortality

## Abstract

**Background:**

It is useful to document whether each newly dominant SARS-CoV-2 variant of concern was more or less dangerous than preceding dominant variant(s). We assessed if the emergence of the Alpha (B.1.1.7) variant in autumn 2020 could be linked to higher case fatality rates, compared to original wild-type COVID-19, subgrouping by age band, sex, deprivation or month of diagnosis as potential risk factors.

**Methods:**

Observational study and secondary analysis were conducted of SARS-CoV-2 cases diagnosed due to medical need or occupational exposure in an administrative area of Eastern England, UK (base population 1 million), who first tested positive in the period 1 March 2020 to 28 February 2021. Multivariate logistic regression was performed to examine relationships of age group, sex, deprivation group and month of diagnosis with case fatality rates within 28 days of diagnosis. Marginal probabilities for risk of dying were calculated separately for the first two main ‘wave’ periods of the English pandemic.

**Results:**

Older age and male sex consistently raised the risk of mortality in both wave periods. Higher deprivation was linked to mortality risk in the first wave period, but not in the second wave. Mortality decreased over time during the first wave period, but slightly increased over time during the second wave. Cases were younger in the second wave, and median age of the deceased varied little between waves.

**Interpretation:**

The Alpha variant of SARS-CoV-2 did not lead to higher mortality rates for any age, deprivation or sex group, compared to case fatality rates in the early part of the pandemic period.

**Supplementary Information:**

The online version contains supplementary material available at 10.1007/s15010-022-01787-x.

## Introduction

The severe acute respiratory syndrome coronavirus two (SARS-CoV-2) virus causes the respiratory illness coronavirus disease (COVID-19) which achieved global pandemic status on 11 March 2020 [1]. COVID-19 is thought to have an all-age case fatality rate between 0.2 and 1.5% [2–4] in high-income countries. It was clear early in the pandemic that mortality and disease severity were strongly dependent on patient age [5]. Treatment strategies rapidly improved early in the United Kingdom (UK) pandemic, leading to higher survival rates [6]. It has not been clear if treatment strategies continued to improve in late 2020/early 2021 or led to better patient outcomes. Early evidence suggested that areas with higher socioeconomic deprivation supplied more cases and relatively more cases with severe outcomes [7]. By late 2020, the concern was also raised that COVID-19 was becoming more dangerous to more segments of the population, following identification of newer variants of SARS-CoV-2, which appeared to be much more transmissible (than earlier recognised variants), especially among relatively younger persons [8].

This article describes a secondary analysis of data that described persons who tested positive for SARS-CoV-19 using reverse transcription polymerase chain reaction tests within a contained region of Eastern England. There were two distinct early ‘wave’ periods when cases and mortality peaked in the UK including in this area of Eastern England. The first wave was marked by great uncertainty about best treatments for COVID-19 patients, while the second wave was affected by an emergent variant of concern (VOC) 202,012/01, formally designated as B1.1.7, also designated by the World Health Organization as the Alpha variant of SARS-CoV-2. B1.1.7 became the predominant SARS-CoV-2 variant in England in early 2021, as tracked by genomically sequenced samples including in our study area. The dataset we analysed refers to only cases detected under the “Pillar 1” testing framework, rather than through community surveillance or any other testing programmes. Age, sex and residential origin area information were available for COVID-19 patients within our study area who had either or both medical need and/or occupational exposure risk factors (“Pillar 1” patients). This dataset allowed us to describe the demographic profile of Pillar 1 COVID-19 patients in this predominantly rural area and compare how much (if at all) the demographic profiles of patients and/or cases who died changed between wave periods, with regard to sex ratio, age distribution, deprivation levels or month of diagnosis.

## Materials and methods

### Data

The dataset described COVID + patients with medical needs or occupational exposure (Pillar 1 status, described in more detail below) treated within the English county of Norfolk and a single district (Waveney) in the adjacent county of Suffolk. Only Pillar 1 cases are described in the analysis in this article, not cases found under any other testing framework. Provision of health care in this combined area was concurrently under the commissioning powers held by the Norfolk and Waveney clinical commissioning group (NWCCG). NWCCG was only permitted to receive and share records of patients registered with NWCCG general practice surgeries. Norfolk and Waveney is a coastal and predominantly rural area in Eastern England, UK that extends approximately 40 × 55 miles. The population is approximately 1 million. The median age of Norfolk residents is 46 years which compares to a median age ~ 40 years for all UK residents in mid-2018 [9]. Comparisons elsewhere showed that Norfolk and Waveney is fairly representative of rural areas of England (UK) with regard to population deprivation indicators, air quality, road network access to employment centres and population density [10]. Permission to analyse these records for epidemiological purposes was granted by our Faculty of Medicine and Health Sciences Ethics Research Ethics Committee, their reference 2019/20–127.

The supplied dataset comprised 8784 unique records of patients who had COVID-19 Pillar 1 positive test results and who received a + COVID Pillar 1 test as reported by National Health Service (NHS) Trusts in the Norfolk and Waveney administrative region through 31 March 2021. Supplementary Table 1 lists the NHS Trusts who provided Pillar 1 records to NWCCG. Case counts and 28-day mortality outcome data were available complete for all patients in all trusts through 31 March 2021 except for the Queen Elizabeth Hospital (QEH); the QEH data feed was unavailable after 17 Feb 2021. Therefore, with regard to mortality outcomes among QEH patients, we ignore cases identified after 17 January 2021. This truncation point allows for 28-day mortality outcomes plus 3 extra days in case of late recording. Partial omission of the QEH data does not bias our analysis of relative risk factors for mortality outcome, because the QEH data contribution is relatively small and we focus on outcomes after diagnosis, rather than outcomes with full population as denominator. Cases detected under the Pillar 1 framework were tested for possible COVID-19 because of medical need for urgent treatment or occupational exposure [11]. The dataset did not distinguish those tested for medical treatment needs from people with occupational risk. We believe most of the records relate to persons with medical need, because 56% of the records were for persons age 65 or older, beyond the recent average age of retirement in England [12], while 74% of the records were for persons age 50 +. The data were collected, cleaned and provided to us by NWCCG. The dataset generally reported which NHS Trust requested the test, residence area resolved to lower super output area (LSOA) geography, age, sex, date that COVID-positive swab was taken and date of death when applicable.

All patients had recorded the date of their + swab test. Information about home residence area for each COVID + patient was available for most records (85%), resolved to LSOA. LSOAs are standard census units in England for which socioeconomic and other indicators are often calculated. LSOAs are designed to be fairly consistent in population but not geographic size. LSOAs typically each contain about 650 households [13]. For each LSOA we accessed the Index of Multiple Deprivation 2019 score (IMD2019) [14]. The IMD2019 is a nationally standardised ranking of relative deprivation, which were categorised here as quintile ranks. Each quintile contains 20% of all LSOAs in England, with most deprived rank = 1 to least deprived rank = 5.

We used only the data records that were complete for all of these attributes: age, sex and residential area. Supplementary Table 1 lists the full number of records received and the number of records that contributed to the descriptive summaries reported in this article.

### Analysis

The data were analysed in two separate time periods. The first period with high case counts (“wave”) was considered to comprise cases swabbed in the period from 1 March to 31 May 2020 inclusive. The second wave was considered to include cases in the period (inclusive) 1 October 2020 to 28 Feb 2021. The dataset was extracted on 8 April 2021 to allow for delayed reporting of outcomes. There were 1997 unique Pillar 1 cases in the first wave, and 6388 unique Pillar 1 cases in the second wave.

We characterise the N and W Pillar 1 populations with regard to demographic traits (age and sex) and deprivation profile over time and between waves. Given the emergence of variants of concern in the UK in the autumn of 2020 [15], and the initially unclear implications of new COVID-19 variants for patient outcomes [8], we were interested in whether the age, sex or deprivation profiles in cases or deaths substantially differed between the two wave periods. Norfolk was identified using genomic analysis by end December 2020 as having a relatively large proportion of cases that were coronavirus novel variant of concern B1.1.7 by the middle of the second wave period [see Supplementary Fig. 1; B.1.1.7 variant comprised 45% of all community samples sequenced in Norfolk in December 2020; 16].

We were interested in assessing risk factors linked to mortality outcomes within this patient group, and whether the mortality risk factors changed over time. Some concurrent national data suggested declining mortality rates among hospitalised COVID-19 patients in the second wave period, and that these improvements were greatest for persons age 70 + (see Supplementary Fig. 2). We restrict the mortality analysis to deaths that occurred no later than 28 days after + swab date, which is appropriate and in line with concurrent national practice about identifying COVID-attributable deaths in the absence of specific causes of death that may be stated on death certificates [17]. Concurrent causes of death on death certificates for N&W in this period were not available to us. We expected that the cases and deaths would both be strongly skewed towards males and older adults. We therefore consider the proportions of the cases and deaths that were male, adults in specific age bands (< 40 years old, 40–49, 50–59, 60–69, 70–79, and 80 +) and in specific deprivation quintiles in the NWCCG area. Possible differences in the age distributions of cases or deceased were formally tested using the Mann-–Whitney *U* test.

We assessed mortality outcomes within 28 days of + swab date using multivariable logistic regression. From these models we also report marginal probabilities of dying linked to each specific risk factor or exposure level, with 95% confidence intervals. Significance was set at *p* ≤ 0.05. The models and linked probabilities were compared descriptively between waves 1 and 2. Models were constructed and analysis was undertaken using Stata 16 and 17.

## Results

**Fig. 1:**
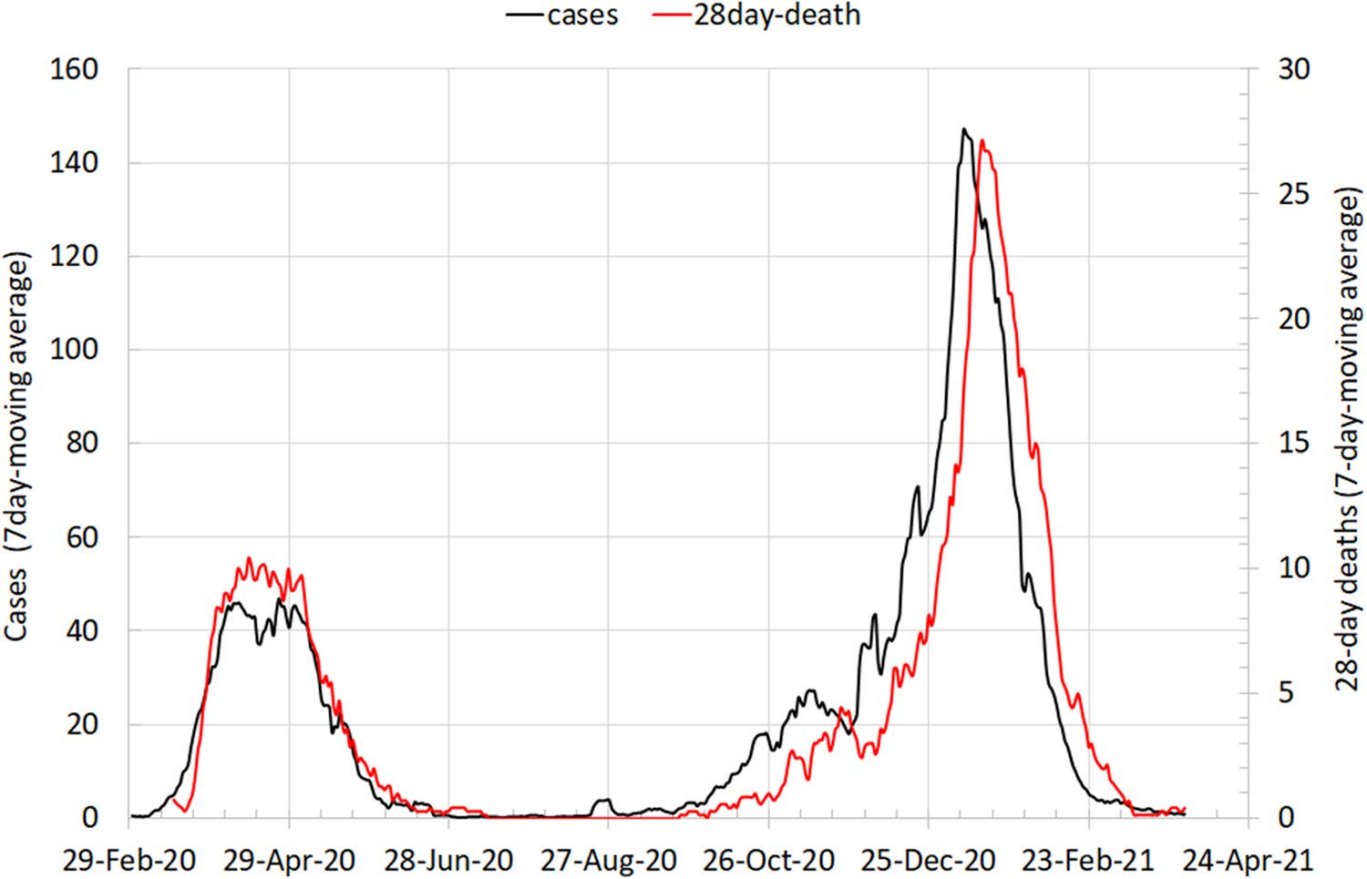
Epidemic curve 29 Feb 2020 to 17April 2021

Figure 1 shows the epidemic curve (case counts) for both waves, presented as 7-day moving (rolling) averages of raw totals. Cases in April 2020 accounted for more than the 50% of cases of the first wave. The second wave was bigger (6389 cases compared to 1997 in Wave 1) and lasted longer (5 months rather than 3 months). Cases diagnosed in January 2021 comprised 49% of second wave cases. Unadjusted case fatality rates are shown by subgroup (for age group, sex, deprivation group or month of diagnosis in Supplementary Table 2).

Table 1 shows demographic traits of the cases and deaths. There were more females diagnosed, but more males who died. Cases tended to be older adults (74% were age 50 +) rather than younger persons, and this age disparity was even more pronounced for mortality outcome (85% of all deaths within 28 days of + swab were among adults age 70 +). Persons residing in the most deprived quintile areas were particularly over-represented as cases in the first wave, and observed to be much more likely to die in the first wave than persons from the least deprived areas. This inequity with regard to deprivation was not apparent in adjusted models describing second wave mortality risk factors.

**Table 1:** Demographic characteristics of the Norfolk and Waveney CCG patient records, and death outcomes within 28 days of a + swab, Pillar 1 cases

	Wave 1			Wave 2	
	Cases *N* (%)	Deaths w/i 28d *N* (% deaths)		Cases *N* (%)	Deaths w/i 28d *N* (% cases)
All	1997	462 (23.1)	All	6389	1097
Sex			Sex		
Male	894 (45)	268 (58)	Male	3211 (50)	593 (54)
Female	1103 (55)	194 (42)	Female	3178 (50)	504 (46)
Age (years)			Age(years)		
< 40	332 (16.6)	5 (1.1)	< 40	1318 (20.8)	4 (0.4)
40–49	159 (8.0)	2 (0.4)	40–49	609 (9.6)	14 (1.2)
50–59	258 (12.9)	18 (3.9)	50–59	769 (12.1)	52 (4.6)
60–69	199 (10.0)	38 (8.2)	60–69	686 (10.8)	106 (9.3)
70–79	302 (15.1)	101 (21.9)	70–79	1034 (16.3)	254 (22.3)
80 +	747 (37.4)	298 (64.5)	80 +	1917 (30.3)	709 (62.2)
Age—median (IQR)	72 (50–85)	83 (75–89)	Age median (IQR)	66 (44–82)	83 (75–89)
IMD2019 quintile			IMD2019 quintile		
1	309 (15.5)	82 (17.7)	1	1043 (19.4)	201 (18.0)
2	492 (24.6)	134 (29.0)	2	1330 (24.8)	262 (23.5)
3	628 (31.4)	155 (33.5)	3	1564 (29.1)	351 (31.5)
4	358 (17.9)	68 (14.7)	4	872 (16.2)	193 (17.3)
5	210 (10.5)	23 (5.0)	5	562 (10.5)	107 (9.6)
Date + swab			Date + swab		
March	266 (13.3)	99 (21.4)	October	345 (5.4)	40 (3.5)
April	1118 (56.0)	275 (59.6)	November	687 (10.7)	101 (8.9)
May	613 (30.7)	88 (19.0)	December	1733 (27.1)	296 (26.0)
			January	3137 (49.1)	599 (52.6)
			February	487 (7.6)	103 (9.0)

First wave + swabs were from 1st March to 31st May 2020, and second wave swabs were those of persons who had + swab in the period inclusive 1 Oct 2020–28 Feb 2021. Deaths w/i 28d means deaths ≤ 28 days after + swab. %s in deaths column are out of total deaths allocated to attribute (eg., sex)

Table 2 shows median age statistics by month over the monitoring period. Median age of persons who presented as cases seems somewhat younger in the second wave, while those who died within 28 days of + swab did not noticeably change from March 2020 to March 2021. This is formally confirmed using Mann–Whitney *U* test to compare case ages from each wave. The Mann–Whitney *U* test comparison for ages of cases between waves was *p* < 0.001, while comparing the age of deceased persons between waves resulted in *p* = 0.215 for the Mann–Whitney *U* test. These results suggest that younger persons were (at *p* ≤ 0.05) significantly more likely to be diagnosed in later months, but there was not a significantly higher risk of younger persons dying within 28 days of diagnosis in the second wave.

**Table 2:** Median age of Pillar 1 cases and deceased by month when Pillar 1 patient had + swab: March 2020 – Feb 2021

Statistic \ month	Mar ‘20	April	May	Jun-Sept	Oct	Nov	Dec ‘20	Jan ‘21	Feb ‘21
Median age (years; all cases)	76	68	73	55	65	67	65	70	64
Median age (years; all deaths within 28 days)	81	84	84	80	81	83	83	82	84
% all cases who died ≤ 28 days	37.2%	24.6%	14.4%	9.9%	12.3%	17.3%	19.9%	19.1%	21.1%

%All cases died within 28 days is with reference to the cases that were swabbed in the indicated month rather than the month in which they died

Table 3 summarises the analyses of the multivariable logistic regression, relating potential risk factors to mortality outcome. Results are broadly similar between the models for each wave with regard to the relative importance of sex, age, deprivation quintile and month of diagnosis. Patients aged 80 + have an increase about 40–200 times higher of death over patients younger than 50 years. Male gender was associated with an approximate 30–45% increase in the odds of dying compared with female. The risk of 28-day death was not different at our pre-specified level of significance (*p* < 0.05) for deprivation in the second wave (*p* = 0.1665 for between group differences on Index of Multiple Deprivation 2019).

**Table 3:** Model coefficients: multivariable logistic regression for the probability of dying within 28 days of a COVID-19 diagnosis, waves 1 and 2

	Wave 1	Wave 2
28-day death	OR (95%CI)	OR (95%CI)
Sex		
Male	1 (Ref.)***	1 (Ref.)***
Female	0.55 (0.4–0.7)	0.64 (0.6–0.7)
Age (vs < 40)	1 (Ref.)***	1 (Ref.)***
40–49	0.73 (0.14–3.8)	7.64 (2.1–27.3)
50–59	4.28 (1.6–11.7)	22.32 (6.9–72.1)
60–69	12.37 (4.7–32.4)	57.72 (18.2–183.3)
70–79	26.29 (10.4–66.4)	96.81 (30.8–304.2)
80 +	42.06 (17.1–103.5)	203.4 (65.0–636.3)
Date swab^a^		
March, October	1 (Ref.)***	1 (Ref.)*
April, November	0.80 (0.6,1.1)	1.40 (0.9–2.2)
May, December	0.35 (0.2–0.5)	1.86 (1.2–2.8)
–, January		1.78 (1.2–2.6)
–, February		1.97 (1.2–3.2)
IMD		
1st quintile	1 (Ref.)**	1 (Ref.)
2nd quintile	0.84 (0.6–1.2)	0.84 (0.7–1.1)
3rd quintile	0.66 (0.5–0.9)	0.89 (0.7–1.1)
4th quintile	0.52 (0.3–0.8)	1.11 (0.9–1.4)
5th quintile	0.40 (0.2–0.7)	0.99 (0.7–1.3)
*N*	1997	5173

First deprivation quintile (IMD) is the cohort living in the 20% most socially deprived areas

^a^*p* < 0.10, *p < 0.05, **p < 0.01, ***p < 0.001

Adjusted for other covariates, marginal probabilities of dying linked to each specific risk factor or exposure level, with 95% confidence intervals are shown for each wave period in Supplemental Figs. 3, 4. These figures are useful for showing the relative importance of each posited risk factor for mortality outcome. Age was the dominant risk factor in both waves, followed by sex and otherwise by month of diagnosis and sometimes deprivation in residence area.

## Discussion

Similar to findings on other cohorts, the greatest risk factor for case status or mortality following COVID-19 diagnosis was advanced age. The NWCCG cohort had somewhat higher case fatality rates than reported elsewhere for persons age 50 +. For instance, we observed a raw case fatality rate (CFR) at about 39% for over 80s in both waves (Supplementary Table 2), while other research in high-income countries suggested a more typical CFR for this age band in the period ending May 2020 might be 29.6% [18]. Our higher CFR probably reflects that these were Pillar 1 cases: most were individuals known to require medical attention at point of diagnosis. Male sex was an expected risk factor for raised mortality, roughly doubling likelihood of death following COVID-19 diagnosis, in line with other observations [19]. Persons living in relatively more deprived areas (IMD quintiles 1–3 were over-represented (relative to the total NWCCG population in these deciles) among both the first wave cases and deaths (Table 1). This conforms with early reporting in England, which found higher COVID cases and mortality in the poorest residential areas [20]. However, in NWCCG data, case fatality rates were much more equitable between deprivation quintiles in the second wave period (Supplementary Table 2) and this is reflected in no significance between deprivation group mortality risk shown in the adjusted mortality model (Table 3). CFRs generally fell during the March–May 2020 period, but there was no consistent chronological trend in CFRs during the months comprising the wave 2 period.

The significance of the contribution of most risk factors for case status or mortality in the first and second waves in Norfolk and Waveney did not differ between waves. The exception was relative deprivation, in that those in the most deprived quintile were much more likely to die following diagnosis in the first wave than they were in wave 2, compared to persons in less deprived quintiles.

We have no specific data to explain why deprivation was much less relevant to death risk (in adjusted models) in the second wave than in the first wave. That persons residing in more deprived areas were more prone to COVID mortality was noted early in the pandemic [7, 21], but the relative contributions of concurrent risk factors are harder to ascertain. Key worker occupations are more prevalent among persons who live in deprived areas rather than affluent areas; it may be that regular testing and more effective social distancing measures were much more relevant to protecting residents of the most deprived areas in the second wave.

The median ages of cases but not deaths (Table 2) tended to be younger in the second wave, which may again reflect more accessible testing rather than more severe disease being found in younger persons. Declining case fatality rates in the first wave over time (Table 2) seem likely to reflect improvement in treatment regimes and agree with other national data on patient outcomes (Supplementary Table 2), but there is some indication of a slight rise in mortality risk during the second wave (*p* = 0.0104 between month difference in Table 3, and marginal probabilities shown in Supplemental Fig. 4). That there were more younger cases in the second wave, but little change in age distribution of the deceased, may also suggest that improvement in treatment methods had the greatest benefits for younger persons.

Unpublished research by others suggested that both the Alpha and Gamma (Pango lineage P.1) SARS-CoV-2 variants were linked to greater illness and hospitalisation of relatively younger persons, compared to original wild COVID [22]. Our data did not indicate that predominance of the Alpha variant in the second wave led to more deaths or hospitalisations of relatively younger persons in NWCCG data, compared to the age distribution seen in the first wave period when no variants of concern had yet been identified. NWCCG Pillar 1 patients did tend to be relatively younger after the first wave (younger median age and younger IQR, Table 2). However, we cannot exclude the possibility that either increased disease suspicion or wider availability of PCR tests made it more likely that relatively less ill patients were identified as Pillar 1 cases after May 2020.

### Effects of UK vaccination programme

We expected the UK vaccination programme to potentially change the age-related risks for mortality following COVID-19 diagnosis. However, determining when an age-related change in mortality statistics might be observable is complex. Community vaccination against SARS-CoV-2 infection began in the UK on 8 December 2020. By 15 February 2021, all health or social care workers and persons age 70 + had been offered a first dose of one of the licensed SARS-CoV-2 vaccines in England [23]. Exactly when vaccination might reduce COVID-19 mortality depended on the speed of the vaccination rollout, which groups received the vaccine first, time elapsed for immune system response, likely delay from exposure to any mortality and social distancing behaviours following vaccination.

Other analyses have found little evidence of immune system protection before 10–12 days post-vaccination, but definite reduced risk of severe illness by 14 days after a single vaccination dose, depending on the specific vaccine product. The estimated risk reductions for severe disease following single doses of the vaccines used in the UK through February 2021 was high, at 66.7% [Vaxzevria; 24] and 90% [Pfizer/BioNTech COVID-19 BNT162b2 vaccine: 25].

The median latent period for SARS-CoV-2 is about 5 days [26]. Of those in our NWCCG cohort who died following Pillar 1 COVID + swabs, 37% died by day 7 afterwards, 50% by day 10 and 80% by day 24. Hence, we observe that most within-28 day mortality happened by day 14 following + swab. Vaccination in the highest risk groups (age 70 +) in NWCCG did not exceed 50% before early February 2021 (see Supplementary Fig. 5). Immediate (within 3 weeks after first vaccine dose) reductions in social distancing precautions were reported by about 40% of persons age 80 + [27]. Consequently, given the vaccine programme start date, likely delay times to mortality outcomes and common behavioural changes post-vaccination, we expect any reduction in mortality from COVID-19 in the NWCCG dataset to not be ascertainable within or before January 2021 data. Fewer cases and subsequent deaths in persons age 70 + might be possible from the start of February 2021, but would be much more confidently expected in March 2021 and later (after our wave periods ended).

### Limitations

Our dataset did not contain information about the ethnicity of patients. Ethnic diversity is quite low in N and W, especially among older adults who are most at risk of severe illness or death from COVID-19 (age 65 +). 96.5% of all-age Norfolk residents self-identified as ‘White’ in the 2011 national census [28]. Assessing ethnic diversity as part of characterising the N and W waves was unlikely to be informative.

Comorbid diagnoses such as diabetes or dementia were available for some but not all patients in our dataset. It is possible that there were changes in case identification or mortality over time or between waves that we did not find, but could have been detected with more complete and detailed individual patient data. Our comparisons between waves are descriptive rather than quantitative precisely because of the lack of prognostic indicators (such as baseline morbidity). There is debate about under-ascertainment of COVID + deaths due to lack of testing and misattribution, especially in the first wave period; we do not have data to assess if our sample was biased with this problem. Similarly, it has been reported [29] that hospitalisations of relatively younger persons were more common in the second UK COVID-19 wave than in March–May 2020. The exact dates of hospitalisation for individual patients were not available in our dataset so we could not consider risk of hospitalisation.

## Conclusion

Within NWCCG, there was higher case detection in younger age cohorts over time. There was decreased mortality over time in the wave one period, but not subsequently over time in wave two. Increasing predominance of the VoC B.1.1.7 from December 2020 onwards did not lead to higher mortality among younger age groups in NWCCG patients. In adjusted logistic regression, residing in a more deprived area increased mortality risk much less in the second wave than it did in the first wave. Advanced age and male sex continued to be the most important risk factors for 28-day mortality throughout the monitoring period.

## Supplementary Information

Below is the link to the electronic supplementary material.

Supplementary file1 (DOCX 300 KB)
