# Supplementary material for: Was Alpha deadlier than wild-type COVID? Analysis in rural England
Source: Infection. 2022 Mar 5;50(5):1171–8. doi: 10.1007/s15010-022-01787-x (PMC8898029; doi:10.1007/s15010-022-01787-x)
Supplement: Supplementary file 1 — Supplementary file1 (DOCX 300 KB) [file 15010_2022_1787_MOESM1_ESM.docx]

# Supplementary Material

## **Supplementary Table 1**. NHS Trusts that provided Pillar 1 test results to NWCCG.

| **Acronym** | **Complete name** | **Principle types of health care services** | **#records**  **start (full_data)** |
| --- | --- | --- | --- |
| ECCH | East Coast Community Health Care | Community hospitals that provide care procedures beyond remit of general practice | 30 (16) |
| JPUH | James Paget University Hospital | Urgent, emergency or consultant-led secondary care | 1010 (866) |
| NNUH | Norfolk and Norwich University Hospital | Urgent, emergency or consultant-led secondary care | 3582 (3549) |
| NSFT | Norfolk and Suffolk Foundation Trust | Mental health and dementia care services | 246 (87) |
| NCHC | Norfolk Community Health and Care | Community hospitals that provide care procedures beyond remit of general practice | 1091 (946) |
| Other | Other NHS bodies | Mostly general practice, primary care | 1854 (1027) |
| QEH | Queen Elizabeth Hospital | Urgent, emergency or consultant-led secondary care | 1305 (1292) |
| WSH | West Suffolk Hospital | Urgent, emergency or consultant-led secondary care | 174 (0) |
|  |  |  |  |

Note: #records start refers to total unique patient records for +Covid swab in period 1 March 2020 to 28 Feb 2021 (last swab date 17 Feb 2021 in case of QEH). The data refer to patients registered at NWCCG primary care practices. Some unique patient records were linked to more than one provider and are double counted in this table. #records (full data) means count of records that had complete data for all of age/sex/deprivation/mortality outcome. Records from WSH provider were incomplete due to lack of home area (postcode) thus no deprivation indicator. The only large provider group with many incomplete records was the ‘Other’ category which reported on 388 Pillar 1 patients in Wave 1, and 1448 patients in Wave 2; this provider category was subsumed into other providers after 28 Jan 2021.

## Supplementary Figure 1. Increased predominance of variant B.1.1.7 (Alpha variant) of SARS-CoV-2 over second wave period


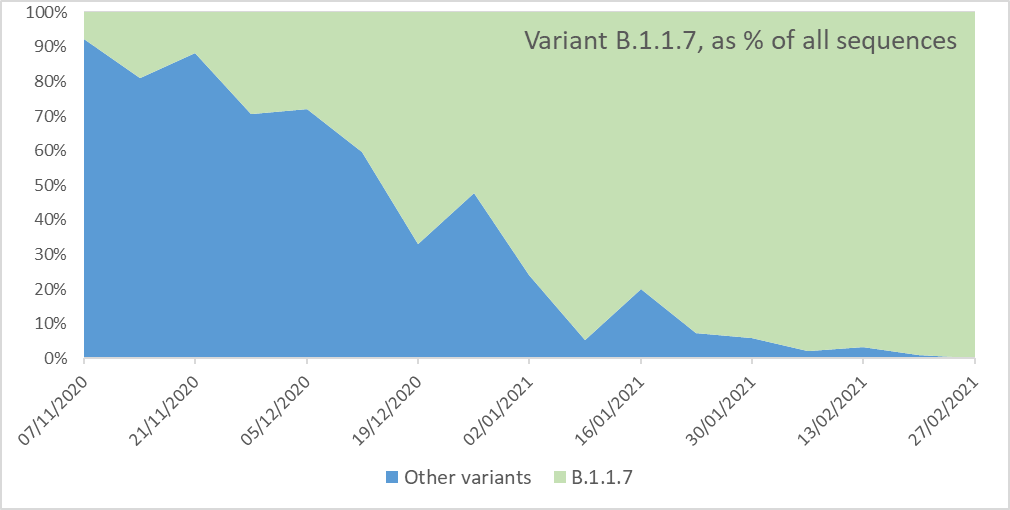


**Source**: Data downloaded from https://covid19.sanger.ac.uk/lineages/raw. Note: Data shown relate only to cases resident in N&W CCG local tier authority areas.

## Supplementary Figure 2. Morality among Covid+ patients following stays in critical care units (all UK)

**Source**: Data updated through 5 July 2021, from the Intensive Care National Audit & Research Centre (ICNARC), https://www.icnarc.org/our-audit/audits/cmp/reports.

## Supplementary Table 2

Case fatality rates (pillar 1 cases) in Norfolk and Waveney (adjusted by the Norfolk and Waveney population total in each subgroup)

|  | Wave 1 | Wave 2 |
| --- | --- | --- |
|  | % (95%CI) | % (95%CI) |
| ALL | 10.7 (9.3-12.2) | 8.3 (7.8-8.9) |
| **SEX** |  |  |
| Male | 11.9 (10.4-13.6) | 9.3 (8.5-10.1) |
| Female | 9.9 (7.3-13.2) | 7.4 (6.6-8.2) |
| **AGE band (yrs)** | |  |
| <40 | 1.9 (0.5-6.2) | 0.2 (0.1-0.7) |
| 40-49 | 1.1 (0.3-4.2) | 2.6 (1.5-4.4) |
| 50-59 | 7.3 (4.7-11.3) | 6.7 (5.1-8.7) |
| 60-69 | 19.1 (14.2-25.3) | 15.7 (13.1-18.7) |
| 70-79 | 33.3 (28-39) | 23.6 (21-26.5) |
| 80+ | 40.3 (36.5-44.2) | 37.3 (34.9-39.7) |
| **IMD quintile** |  |  |
| 1 | 18.5 (15.6-21.7) | 9.1 (7.8-10.5) |
| 2 | 12.1 (10-14.7) | 8.3 (7.2-9.6) |
| 3 | 12.6 (10.4-15.2) | 8.4 (7.3-9.5) |
| 4 | 7.4 (5.3-10.2) | 9.5 (8.1-11.1) |
| 5 | 4.2 (2.6-6.7) | 8.1 (6.4-10.2) |
| **DATE +swab** | |  |
| March 2020 | 24.2 (19.8-29.1) |  |
| April 2020 | 11.2 (9.7-12.9) |  |
| May 2020 | 5.9 (4.5-7.8) |  |
| October 2020 |  | 6.3 (4.3-9.1) |
| Nov. 2020 |  | 6.6 (5.2-8.3) |
| Dec. 2020 |  | 8.9 (7.8-10.2) |
| January 2021 |  | 8.8 (8-9.7) |
| Feb. 2021 |  | 7.2 (5.4-9.6) |

Notes: CFR = Case fatality rate. CFRs were adjusted by Norfolk and Waveney population totals in each subgroup. The 95% intervals are calculated as binomial CI.

Supplementary Figure 3: Marginal probabilities of dying in the CCG dataset after multivariable logistic regression: Wave 1

**
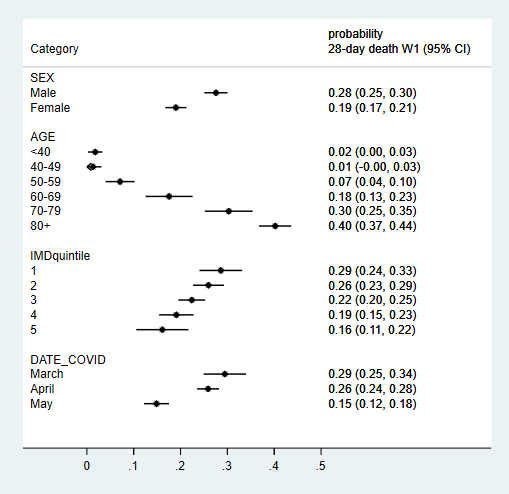
**

*Note*: the probabilities shown were calculated only with respect to all cases in the Pillar 1 dataset analysed in the main manuscript.

## Supplementary Figure 4: Marginal probabilities of dying in the CCG dataset after multivariable logistic regression: Wave 2


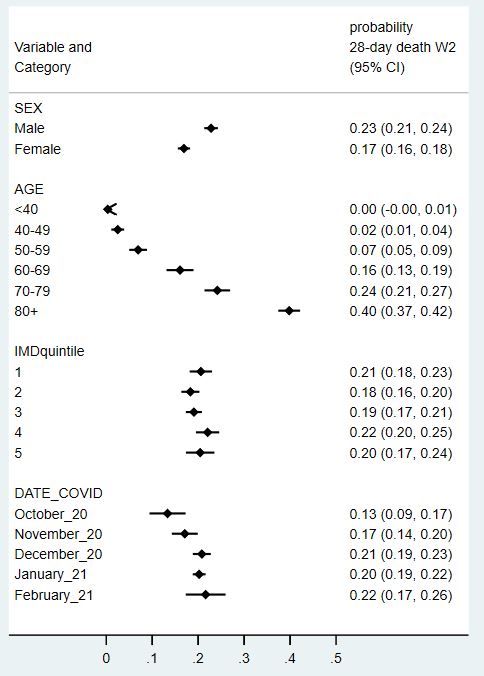


*Note*: the probabilities shown were calculated only with respect to all cases in the Pillar 1 dataset analysed in the main manuscript.

## Supplementary Figure 5. Vaccination uptake in NWCCG in persons age 70+


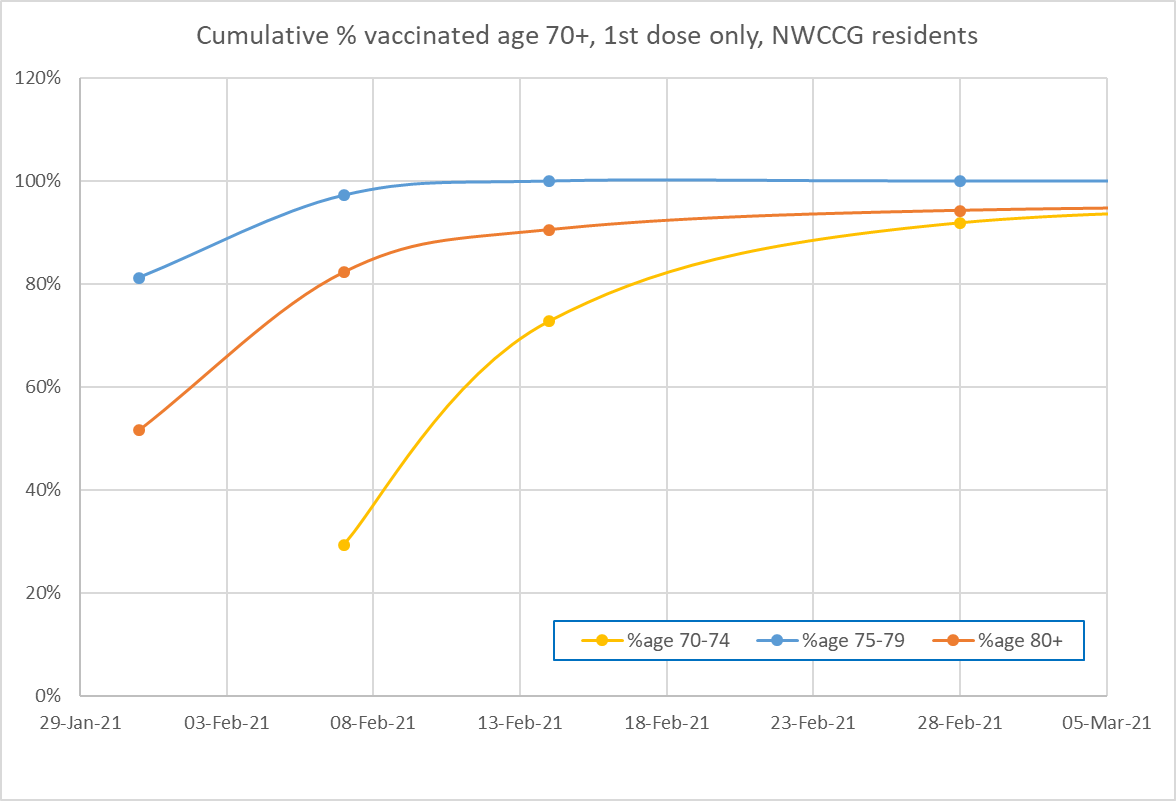


Concurrent priority groups (only groups) eligible for vaccination until 15 February 2021:

1. care home residents and their carers

2. people over the age of 80 and frontline health and social care workers

3. people aged 75 to 79

4. people aged 70-74 and those under 70 deemed to be “clinically extremely vulnerable”

**Source**: Data available at https://www.england.nhs.uk/statistics/statistical-work-areas/covid-19-vaccinations/. Persons age 75+ may have been vaccinated more quickly than persons age 80+ due to greater mobility (better access to transport to vaccine centres, fewer were house-bound).
